# Analysis of the 10q11 Cancer Risk Locus Implicates *MSMB* and *NCOA4* in Human Prostate Tumorigenesis

**DOI:** 10.1371/journal.pgen.1001204

**Published:** 2010-11-11

**Authors:** Mark M. Pomerantz, Yashaswi Shrestha, Richard J. Flavin, Meredith M. Regan, Kathryn L. Penney, Lorelei A. Mucci, Meir J. Stampfer, David J. Hunter, Stephen J. Chanock, Eric J. Schafer, Jennifer A. Chan, Josep Tabernero, José Baselga, Andrea L. Richardson, Massimo Loda, William K. Oh, Philip W. Kantoff, William C. Hahn, Matthew L. Freedman

**Affiliations:** 1 Department of Medical Oncology, Dana-Farber Cancer Institute, Boston, Massachusetts, United States of America; 2 Department of Medicine, Brigham and Women's Hospital and Harvard Medical School, Boston, Massachusetts, United States of America; 3 Center for Cancer Genome Discovery, Dana-Farber Cancer Institute, Boston, Massachusetts, United States of America; 4 Broad Institute of Harvard and Massachusetts Institute of Technology, Cambridge, Massachusetts, United States of America; 5 Center for Molecular Oncologic Pathology, Dana-Farber Cancer Institute, Brigham and Women's Hospital, Boston, Massachusetts, United States of America; 6 Department of Biostatistics and Computational Biology, Dana-Farber Cancer Institute and Harvard Medical School, Boston, Massachusetts, United States of America; 7 Department of Epidemiology, Harvard School of Public Health, Boston, Massachusetts, United States of America; 8 Channing Laboratory, Department of Medicine, Brigham and Women's Hospital and Harvard Medical School, Boston, Massachusetts, United States of America; 9 Department of Nutrition, Harvard School of Public Health, Boston, Massachusetts, United States of America; 10 Division of Cancer Epidemiology and Genetics, National Cancer Institute, National Institutes of Health, Bethesda, Maryland, United States of America; 11 Department of Pathology and Laboratory Medicine, University of Calgary, Calgary, Alberta, Canada; 12 Vall d'Hebron Institute of Oncology, Vall d'Hebron University Hospital, Barcelona, Spain; 13 Department of Pathology, Brigham and Women's Hospital and Harvard Medical School, Boston, Massachusetts, United States of America; 14 Tisch Cancer Institute, Mount Sinai School of Medicine, New York, New York, United States of America; Stanford University School of Medicine, United States of America

## Abstract

Genome-wide association studies (GWAS) have established a variant, rs10993994, on chromosome 10q11 as being associated with prostate cancer risk. Since the variant is located outside of a protein-coding region, the target genes driving tumorigenesis are not readily apparent. Two genes nearest to this variant, *MSMB* and *NCOA4*, are strong candidates for mediating the effects of rs109939934. In a cohort of 180 individuals, we demonstrate that the rs10993994 risk allele is associated with decreased expression of two *MSMB* isoforms in histologically normal and malignant prostate tissue. In addition, the risk allele is associated with increased expression of five *NCOA4* isoforms in histologically normal prostate tissue only. No consistent association with either gene is observed in breast or colon tissue. In conjunction with these findings, suppression of *MSMB* expression or *NCOA4* overexpression promotes anchorage-independent growth of prostate epithelial cells, but not growth of breast epithelial cells. These data suggest that germline variation at chromosome 10q11 contributes to prostate cancer risk by influencing expression of at least two genes. More broadly, the findings demonstrate that disease risk alleles may influence multiple genes, and associations between genotype and expression may only be observed in the context of specific tissue and disease states.

## Author Summary

Family history has long been recognized as an important risk factor for prostate cancer. Beginning in 2006, researchers have identified several genetic variants that are associated with prostate cancer risk. Intriguingly, the majority of prostate cancer risk variants do not reside in genes. Determining the genes involved in the development of disease, therefore, has proved challenging. In this study we interrogate a known prostate cancer risk polymorphism on chromosome 10—rs10993994. We report that this variant is significantly associated with the RNA expression levels of two genes—*MSMB* and *NCOA4*. When these expression changes are modeled in a cell line, prostate cells that were previously non-tumorigenic acquire a property known as anchorage independence, a characteristic of cancer cells. Notably, the prostate risk variant is *not* associated with expression or functional changes in breast or colon cells. In addition, the effects are most pronounced in normal rather than tumor prostate tissue. Overall, these findings help define the genes driving prostate cancer risk at chromosome 10. More generally, the discoveries demonstrate the importance of considering several target genes, as well as the importance of cellular context (tissue type and histological state), in future analyses of other genetic risk regions.

## Introduction

Variation at rs10993994 on chromosome 10q11 is associated with prostate cancer risk [1]–[5]. The risk polymorphism is located at the telomeric end of a 50 kilobase (kb) linkage disequilibrium block and is within 60 base pairs (bp) of the transcription start site of beta-microseminoprotein (*MSMB*). *MSMB* has been characterized as a tumor suppressor [6], and lower levels of its product, PSP94, are associated with more aggressive forms of prostate cancer [7]. *MSMB* has therefore been a target of recent investigation into the mechanism of chromosome 10q-associated risk. Reporter assays demonstrate that plasmids carrying the rs10993994 risk allele (T) significantly decrease luciferase activity compared with the wild-type allele (C) [8], [9]. In addition, in 19 cancer cell lines of various tissue types expressing *MSMB*, those carrying the TT genotype have decreased *MSMB* expression relative to those carrying a C allele [9]. However, no study has definitively linked *MSMB* expression to risk allele status in human prostate tissue. A second gene, nuclear receptor co-activator 4 (*NCOA4,* also known as *ARA70*), is a ligand-dependent androgen receptor co-activator [10], [11] and is within 16 kb telomeric of rs10993994. Given its proximity to the risk variant and its activity in the prostate gland [12], NCOA4 has also been considered a candidate gene involved in the mechanism of disease risk [8].

Gene expression is a heritable trait [13]–[16] and represents a powerful avenue for connecting risk variants with their target genes. Studies have demonstrated that variation at intergenic or intronic disease-associated loci can act through gene regulatory mechanisms [17]–[20]. Because regulatory elements can interact with many genes [21], and since both *MSMB* and *NCOA4* are strong candidates for prostate cancer risk, we evaluated the relationship between risk allele status and transcript abundance of these genes across both normal and tumor prostate tissues. We also tested the functional consequences of altering the expression levels of these candidate genes in immortalized prostate epithelial cells.

## Results

**Figure 1:**
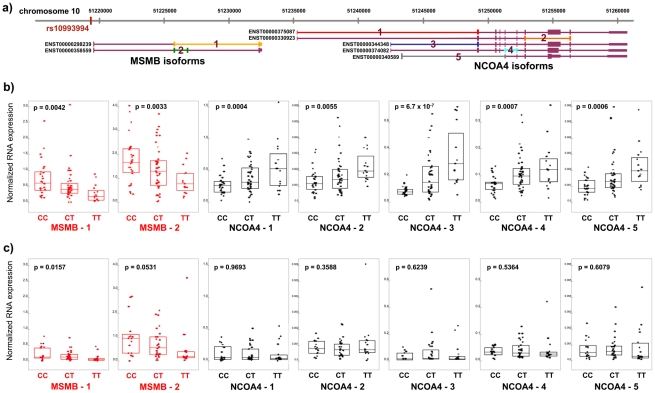
RNA expression of *MSMB* and *NCOA4* in normal and tumor prostate tissue by rs10994994 genotype. A. Chromosome 10q11 with isoforms of *MSMB* and *NCOA4* (Ensembl build 52). Primers for competitive PCR were designed to cross exon-exon boundaries depicted by colored lines 1–2 (*MSMB*) and 1–5 (*NCOA4*). B. Expression in histologically normal prostate tissue (n = 84). Each point represents absolute RNA expression for one individual, normalized to three housekeeping genes. The top and bottom of the boxes within each graph represent the upper and lower quartiles for expression at each genotype. The band inside each box marks the median value. P-value for each graph denotes the significance for association between expression and genotype. C. Expression in prostate tumor tissue in the Dana-Farber Cancer Institute series (n = 61).

A total of 180 individuals of European ancestry were genotyped for the rs10993994 polymorphism, and *MSMB* and *NCOA4* mRNA levels were quantified in tissue isolated from radical prostatectomy surgical specimens. Samples were derived from two cohorts- the Gelb Center at Dana-Farber Cancer Institute (DFCI) (N = 121 – histologically normal and tumor prostate tissue) and the Physicians’ Health Study (PHS) [22] (N = 59 – prostate tumor tissue only). In the DFCI cohort, transcript levels were measured in both normal and tumor prostate tissue using a quantitative competitive PCR strategy (Methods). Two *MSMB* and five *NCOA4* isoforms annotated in the Ensembl database (build 52) were evaluated (Figure 1A). Each isoform of *MSMB* and *NCOA4* was expressed in both normal and tumor prostatic tissue. Expression levels of *MSMB* and *NCOA4* transcripts were significantly higher in normal compared with tumor tissue (p<0.0001), consistent with previously published reports [23]–[25]. In the PHS cohort, only tumor tissue was isolated from radical prostatectomy specimens. For this cohort the probes used to measure expression captured both *MSMB* isoforms and all but one of the *NCOA4* isoforms.

The T (risk) allele is significantly associated with transcript levels of both *MSMB* and *NCOA4* in histologically normal prostate tissue (N = 84). While the T allele is associated with decreased expression of both *MSMB* isoforms (p-value range, 0.0033–0.0042), it is associated with *increased* expression of all five isoforms of *NCOA4* (p-value range, 6.7×10^−7^–0.0055) (Figure 1B). In tumor tissue, *MSMB* retains its association with risk allele status (p-value range, 0.016–0.053, Figure 1C, Figure S1). *NCOA4* expression levels, however, are no longer associated with genotype in tumor tissue (p>0.30, Figure 1C, Figure S1). The associations are specific to the genes in this region. Expression levels of *TIMM23*, the next closest gene to the risk locus and 1.4 kb telomeric to *NCOA4*, are not correlated with genotype status in either normal or tumor tissue. (p>0.30, Figure S2).

Because rs10993994 is *not* a risk allele in colon or breast cancers, we reasoned that the association between genotype and disease-relevant genes may be specific to prostate tissue and not observed in other tissue types. If an association between genotype and expression of *MSMB* or *NCOA4* were observed in tissue other than prostate, then that gene may be less likely to be involved in prostate cancer risk. *MSMB* and *NCOA4* expression levels were measured in histologically normal colon (N = 72) and breast (N = 56) tissue samples. While breast tissue expresses both genes, colon tissue only expresses *NCOA4*. Unlike prostate tissue, neither breast nor colon tissue demonstrates convincing or consistent associations with genotype across isoforms (Figure S3).

**Figure 2:**
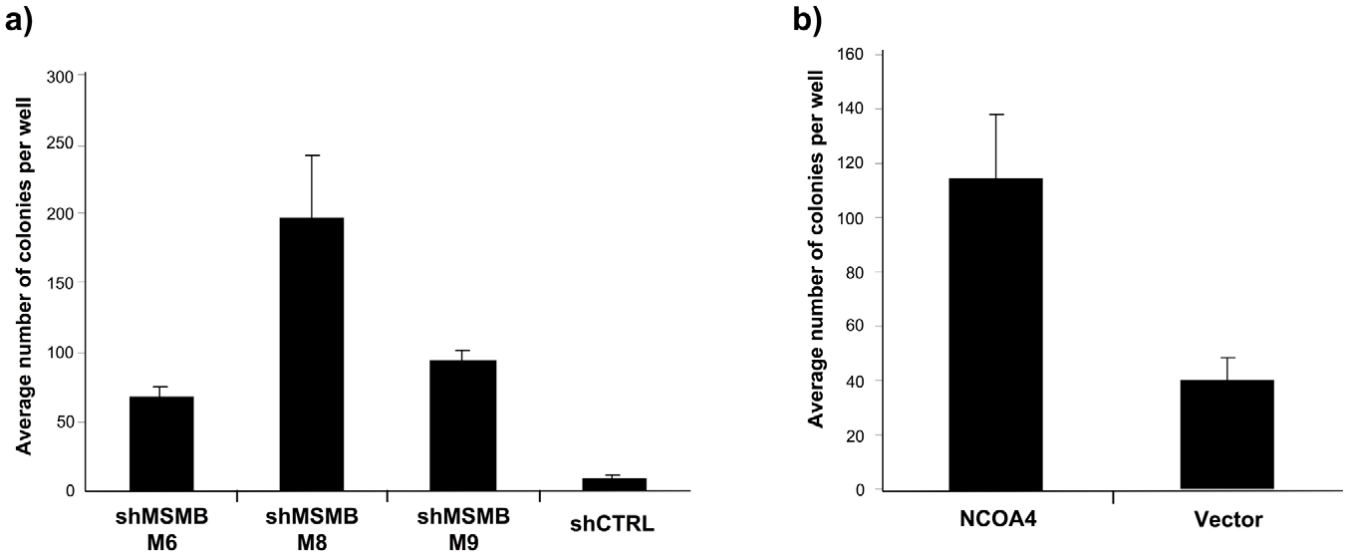
Suppressing *MSMB* or overexpressing *NCOA4* is associated with increased anchorage-independent growth of prostate epithelial cells. A. Effects of suppressing *MSMB* with three independent shRNAs in LHSAR cells (p-values 0.0001; M6, 0.0023; M8, and 0.0001; M9). The increase in anchorage-independent growth inversely correlates with the degree of *MSMB* suppression (Figure S4). B. Anchorage-independent growth of LHSAR cells overexpressing *NCOA4* and a control vector (p = 0.0074).

To evaluate the functional implications of the genetic findings, we tested the effect of increasing *NCOA4* and suppressing *MSMB* expression levels in immortalized prostate epithelial cells (LHSAR) [26]. Specifically, we assessed the ability of *NCOA4* and *MSMB* to promote anchorage-independent growth, a phenotype strongly associated with cell transformation [27]–[30]. Suppression of *MSMB* expression in LHSAR cells led to a significant increase in anchorage-independent colony growth (p-values 0.0023–0.0001; Figure 2A, Figure S4). Overexpression of *NCOA4* in LHSAR cells also resulted in robust anchorage-independent colony growth (p-value 0.0074; Figure 2B, Figure S4). To assess whether these alterations were specific for prostate epithelial cells, similar functional studies were performed in immortalized human mammary epithelial cells [31]. In contrast to what was observed in prostate epithelial cells, manipulating expression levels of *MSMB* or *NCOA4* did not result in any consistently significant change in the anchorage-independent growth in mammary epithelial cells (Figure S5). Together, these observations implicate a role for both *NCOA4* and *MSMB* in the transformation of prostate epithelial cells.

## Discussion

Genetic data presented here demonstrate that the chromosome 10q11 prostate cancer risk locus is associated with decreased levels of *MSMB* and increased levels of *NCOA4* RNA expression. Strikingly, the functional data fully corroborate the genetic data. When *MSMB* is knocked down or NCOA4 is overexpressed in immortalized prostate epithelial cells, the cells become anchorage independent. Our data suggest that both *MSMB* and *NCOA4* mediate prostate tumorigenesis, and this study is the first, to our knowledge, to implicate these genes in actual human prostate tissue. As expected in a cohort comprised of subjects who underwent radical prostatectomy, a large majority of individuals included in the analysis were diagnosed with low- or intermediate-risk prostate cancer. Despite a relatively homogenous cohort, the results presented here are likely generalizable to most prostate cancer cases since rs10993994 appears to confer risk for all levels of prostate cancer aggressiveness [1], [3], [32], [33], [34].

Similar to the 10q11 risk allele, other disease risk loci have been shown to affect expression of more than one gene [17], [34]. A variant associated with systemic lupus erythematous at chromosome 8p23, for example, is associated with increased expression of one gene (*BLK*) and decreased expression of another (*C8orf13*) in B cell lines [34]. As more genes underlying complex traits are discovered, it may be that certain risk alleles mediate their effects through multiple genes, or alternatively, that two risk variants in tight linkage disequilibrium influence separate genes. The findings at 10q11 highlight the importance of considering multiple genes when analyzing GWAS results.

The findings at 10q11 also underscore the importance of evaluating risk loci in a tissue-specific context [35]. It is hypothesized that a fraction of non-protein coding risk alleles will alter disease risk by regulating gene activity, and these variants may exert their effects in a specific genetic and epigenetic context [21], [36]. In the present study, the 10q11 risk variant is associated with transcript levels of *MSMB* and *NCOA4* in primary prostate tissue. In contrast, no convincing or consistent association is observed in colon or breast tissue. Similarly, alteration of *MSMB* and *NCOA4* expression significantly affects anchorage-independence of prostate but not breast epithelial cells. These findings may have implications for future studies attempting to connect risk alleles with target gene(s). Evaluation of GWAS findings will focus, in part, on identifying the genes targeted by risk alleles, as these are the genes likely to drive the trait under study. Our findings suggest that this type of analysis should include evaluation of the tissue at risk for disease, although it is entirely plausible that variants associated with a particular disease may manifest their effects in tissues other than target tissue.

Notably, associations between rs10993994 genotype and expression of *MSMB* and *NCOA4* are observed in histologically normal prostate tissue, whereas in tumor tissue an association is detected with only *MSMB* (albeit at an attenuated level relative to the normal tissue). It is conceivable that increased expression of *NCOA4* is associated with tumor initiation, as reflected by its association with risk in solely normal tissue, while decreased expression of *MSMB* is associated with both tumor initiation and maintenance or progression. More studies, however, will be necessary before a general principle emerges.

Cellular context also appears to be an important determinant in the functional analysis of candidate risk genes. This is illustrated by comparing data in the present study to previous work involving *NCOA4*. The characteristics of two NCOA4 isoforms, alpha and beta, have been studied in functional assays. Upregulation of NCOA4beta increases anchorage-independent growth [11], while overexpression of NCOA4alpha inhibits growth in LNCaP cells [37]. Functional analysis of immortalized prostate epithelial cells presented here demonstrates *increased* colony growth in the setting of an overexpressed alpha isoform (Figure 2). Distinctions between the cell lines used in these studies may account for these divergent results. LNCaP cells are derived from metastatic prostate lesions. Immortalized prostate epithelial cells, on the other hand, are not tumorigenic and differentiate in the presence of androgen [26], suggesting that these cells are more closely related to normal prostate epithelial cells.

The data presented here suggest that tissue type (in this case, prostate versus non-prostate tissue) and cellular states (i.e., normal versus tumor) are likely important factors in the evaluation of complex trait loci. Chromatin context and differential use of gene regulatory elements across tissues and disease states may be the basis for expression effects specific to normal prostate tissue [36]. It can be difficult to accurately model these effects outside of the particular genetic and epigenetic context of specific tissues. Luciferase reporter assays, for example, are often utilized to define a relationship between a polymorphism and a gene. Reporter assays cannot, however, detect situations where a risk variant is associated with opposing transcriptional effects on two loci, as observed with rs10993994. An alternative explanation for the effects on the two transcripts is that two separate variants in linkage disequilibrium are responsible for the different transcriptional effects.

In contrast to Mendelian diseases, where resequencing protein-coding regions often reveals the causal gene, common complex trait alleles often occur outside of protein-coding regions. As is the case at 10q11 and other loci [38], [39], these alleles may be associated with expression of nearby and/or distal candidate genes. There are also situations in which associations with strong candidate genes, however, cannot be established by measuring steady-state expression at a single point in time [19]. In order to better understand the gene(s) involved in complex trait pathogenesis, experiments will need to integrate and to account for the genetic and epigenetic contexts of the particular tissue type and cellular state.

## Materials and Methods

### Ethics statement

This study was conducted according to the principles expressed in the Declaration of Helsinki. The study was approved by the Institutional Review Board of Dana-Farber Cancer Institute. All patients provided written informed consent for the collection of samples and subsequent analysis.

### Cohorts and RNA isolation

A total of 180 patients treated with radical prostatectomy (RP) for prostate cancer and 92 patients treated for colon cancer consented to provide tissue. Additionally, histologically normal breast tissue from 56 from women undergoing cosmetic reduction surgery was analyzed in the present study.

Fresh frozen radical prostatectomy specimens were available from 121 subjects at the Dana-Farber Cancer Institute (DFCI) and Brigham and Women's Hospital (Boston, MA) and were reviewed by a pathologist (J.C. or R.F.). Over 95% of the patients in the cohort were diagnosed with Gleason 6 or Gleason 7 disease and median PSA was 5.1 ng/ml. Areas of tumor consisted of >60% tumor cells and areas of benign tissue consisted of >80% non-neoplastic epithelial cells at least 5 mm away from any tumor focus. Biopsy cores of fresh frozen tissue were processed for RNA extraction using a modified Qiagen Allprep DNA/RNA protocol.

Archival FFPE blocks were available for 59 men with prostate cancer enrolled in the Physicians’ Health Study (PHS) [22], [40]. These men were diagnosed with prostate cancer between 1983 and 2003 and treated by radical prostatectomy. RNA were extracted from paraffin-embedded tumor tissue as described previously [40]. Areas of tumor consisted of >90% tumor cells.

Fresh frozen colorectal cancer tissue samples were reviewed by a pathologist (J.C.) and areas of benign tissue were selected where 80% of cells consisted of non-neoplastic epithelium. RNA was extracted using a modified Qiagen Allprep DNA/RNA protocol. Fresh frozen normal breast tissue samples were reviewed to identify tissue blocks containing >40% normal epithelial cells. RNA was extracted using a modified Qiagen RNeasy protocol.

Ethnicity was self-reported by most, but not all, subjects. Subjects in the DFCI cohort of unknown ancestry were genotyped for 59 ancestry-informative SNPs in order to ascertain ethnicity. The marker set primarily captured ancestral differences between European and African ancestries (D. Reich, personal communication). Five samples found to be from subjects of African ancestry were excluded from analysis.

The human tissues analyzed in this study were from patients treated at Brigham and Women’s Hospital, Dana-Farber Cancer Institute or Vall d'Hebron University Hospital in Barcelona, Spain, all of whom provided informed consent. The study was approved by the Institutional Review Board at Dana-Farber Cancer Institute.

### Expression analysis

cDNA was prepared for expression analysis using Invitrogen SuperScript III Reverse Transcription kit. DFCI prostate samples, colon samples and breast samples were analyzed via competitive RT-PCR using Sequenom iPLEX matrix-assisted laser desorption/ionization (MALDI)-time of flight mass spectrometry technology. Expression levels of two *MSMB* isoforms and five *NCOA4* isoforms were measured. These splice variants represent all isoforms reported in Ensembl genebuild 52. RNA expression of *TIMM23* and three housekeeping genes (ACTB, MYL6 and RPL13A) were also measured. Primer, probe and competitor oligo sequences are available upon request. Reactions were performed in quadruplicate using 8 serial dilutions of competitor, and the EC50 was calculated using QGE Analyzer software (Sequenom). The PHS subgroup was analyzed using Illumina cDNA-mediated Annealing, Selection, Extension and Ligation (DASL) expression assay (Illumina).

### Expression data analysis

A gene expression normalization factor was calculated using the geometric mean of expression level of the three housekeeping genes. Linear regression was used to assess whether expression levels increased (or decreased) as the number of T-alleles of rs10993994 increased; for prostate tissue, differences between prostate tumor and normal tissue levels were also assessed and random effects linear regression was used to account for within-sample correlation of tumor/normal pairs.

### Genotyping

Genotyping of DNA from each subject was carried out using Sequenom iPLEX matrix-assisted laser desorption/ionization (MALDI)-time of flight mass spectrometry technology.

### Cell culture

LHSAR: Prostate epithelial cells (PrECs) immortalized with hTERT, SV40 Large T and small t antigens and overexpressing androgen receptor were grown in PREGM (Lonza CC-3166). HMLE: Human mammary epithelial cells immortalized with hTERT, Large T and small t antigens were grown in MEGM (Lonza CC-3150). All growth media were supplemented with 100 ug/ml Penicillin/Streptomycin.

### Soft agar colony formation assay

The bottom layer contained 0.6% agar (Sigma A5431) in DMEM and 8% FBS. The top layer contained 0.3% agar in PREGM or MEGM for LHSAR or HMLE respectively. Fifteen thousand cells were seeded in the top agar layer in triplicate wells of a 6 well plate. Colonies were counted from 2 to 6 weeks post-seeding. Image of each well was taken at a 6× magnification and analyzed with Image J software. Colonies that were 50 sq. pixels or larger were counted.

### Quantitative RT-PCR

Qiagen RNeasy kit was used for RNA extraction. Reverse transcription was carried out with Clonetech Advantage RT-to-PCR kit while the quantitative PCR was carried out using SYBR Green Master Mix (Applied Biosystems). Two sets of *NCOA4* and one set of *MSMB* primers were used:

1. NCOA4 exon 3-4 - Forward CAGCAGCTCTACTCGTTATTGG
  Reverse TCTCCAGGCACACAGAGACT
2. NCOA4 exon 5-6 - Forward CTCTCAAAACCATTCAAATTCCT
  Reverse CTCTGGCATGGAGATACAGC
3. MSMB exon 1-2 - Forward GCTTATCACAATGAATGTTCTCCT
  Reverse AATCTCCTGGAACTCCCTCA

### Expression constructs

*NCOA4* expression constructs were received from the human ORFeome V5.1 and cloned into pWZL-Neo retroviral expression vector. Retrovirus production, infection and selection were carried out as described previously [41].

### RNA interference

Short hairpins in pLKO.1 lentiviral constructs were received from the RNAi Consortium (TRC). Lentivirus production and infection were carried out as described previously [42].

1. Hairpin shMSMB M6 TRC Clone ID TRCN0000147237
Target sequence GTTCTGTCAGTGAATGGATAA
2. Hairpin shMSMB M8 TRC Clone ID TRCN0000146396
Target sequence CACCTTCGTGACTTTATGCAA
3. Hairpin shMSMB M9 TRC Clone ID TRCN0000146343
Target sequence CAAAGGAAACAAACACCCAAT

## Supporting Information

Figure S1 RNA expression of MSMB and NCOA4 in tumor prostate tissue by genotype at rs10994994. Expression in prostate tumor tissue in the Physicians’ Health Study series (n = 59). Each point represents absolute RNA expression for one individual. The top and bottom of the boxes within each graph represent the upper and lower quartiles for expression at each genotype. The band inside each box marks the median value. P-value for association with MSMB expression, 0.0073. P-value for association with NCOA4 expression, 0.2671.
(0.50 MB TIF)

Figure S2 Expression of TIMM23 at chromosome 10q11 is not associated with genotype at rs10993994 in prostate tissue. A. Expression in histologically normal prostate tissue (n = 84, p = 0.3459). B. Expression in prostate tumor tissue in the Dana-Farber Cancer Institute series (n = 61, p = 0.9939).
(9.46 MB TIF)

Figure S3 *MSMB* and *NCOA4* expression in breast and colon epithelial tissue. Expression of *MSMB* and NCOA4 isoforms are not consistently associated with genotype at rs10993994 in breast or colon epithelial tissue. A. Expression in histologically normal breast tissue (n = 56). P-values for association between genotype and expression included within each graph. B. Expression in histologically normal colon tissue (n = 72).
(9.47 MB TIF)

Figure S4 Quantitative RT-PCR for the expression of MSMB and NCOA4 in LHSAR cells. A. MSMB suppression with three individual shRNAs (shMSMB M6, M8 and M9). B. NCOA4 overexpression.
(9.46 MB TIF)

Figure S5 Anchorage-independent growth in human mammary epithelial cells. MSMB and NCOA4 expression levels do not consistently affect anchorage-independent growth of human mammary epithelial cells (HMLE). A. Suppression of MSMB by three independent shRNAs (p-values 0.0949, 0.7482, and 0.0197). B. NCOA4 overexpression (p = 0.1012).
(9.46 MB TIF)
