# Supplementary figures and images for: Analysis of the 10q11 Cancer Risk Locus Implicates MSMB and NCOA4 in Human Prostate Tumorigenesis
Source: PLoS Genet. 2010 Nov 11;6(11):e1001204. doi: 10.1371/journal.pgen.1001204 (PMC2978684; doi:10.1371/journal.pgen.1001204)

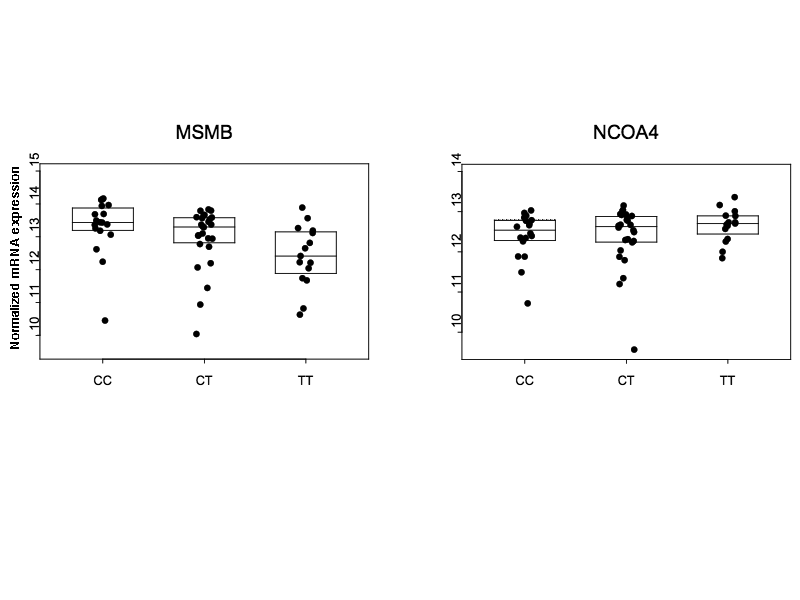

Supplement: Figure S1 — RNA expression of MSMB and NCOA4 in tumor prostate tissue by genotype at rs10994994. Expression in prostate tumor tissue in the Physicians’ Health Study series (n = 59). Each point represents absolute RNA expression for one individual. The top and bottom of the boxes within each graph represent the upper and lower quartiles for expression at each genotype. The band inside each box marks the median value. P-value for association with MSMB expression, 0.0073. P-value for association with NCOA4 expression, 0.2671. (0.50 MB TIF) [file pgen.1001204.s001.tif]

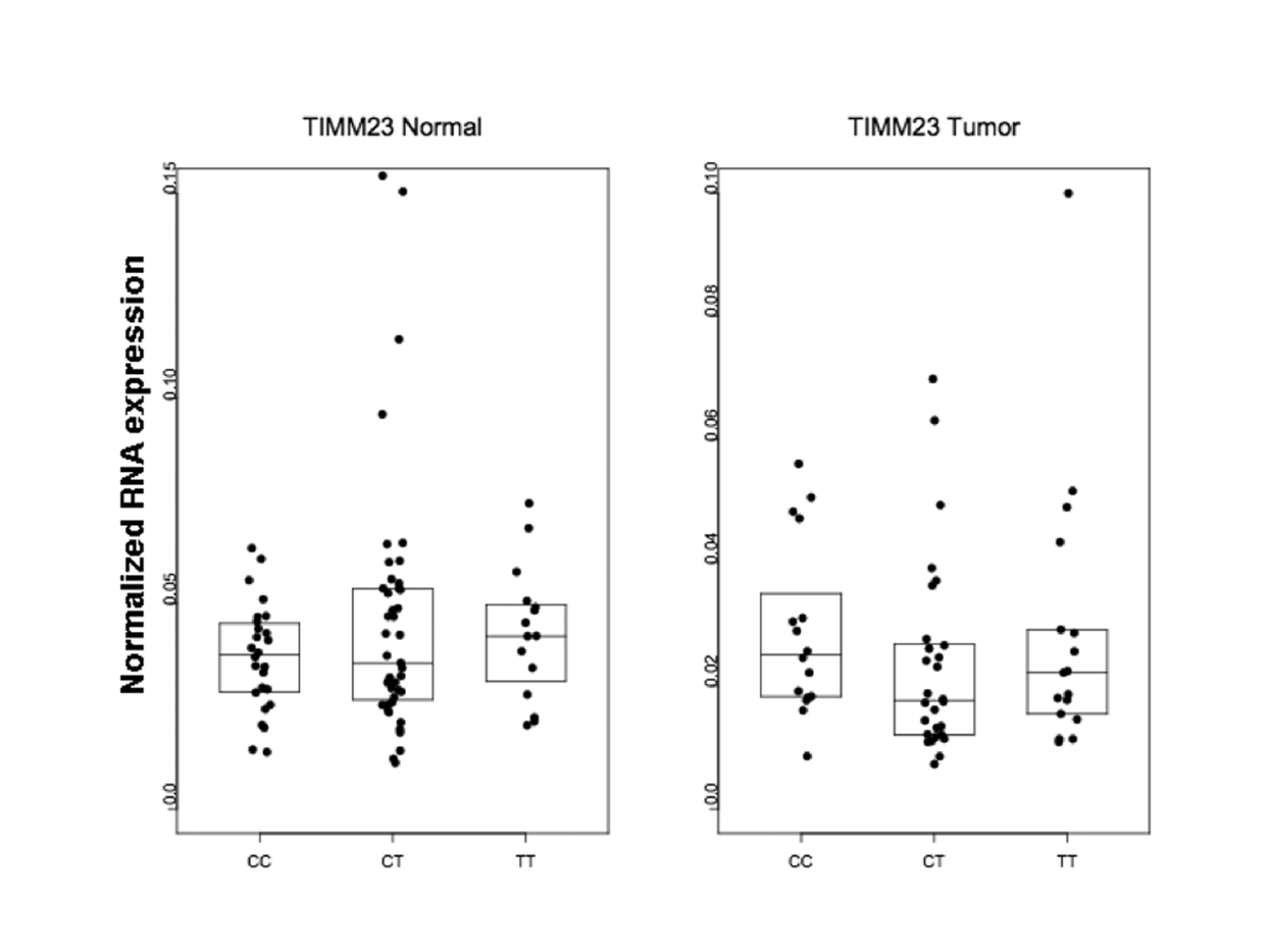

Supplement: Figure S2 — Expression of TIMM23 at chromosome 10q11 is not associated with genotype at rs10993994 in prostate tissue. A. Expression in histologically normal prostate tissue (n = 84, p = 0.3459). B. Expression in prostate tumor tissue in the Dana-Farber Cancer Institute series (n = 61, p = 0.9939). (9.46 MB TIF) [file pgen.1001204.s002.tif]

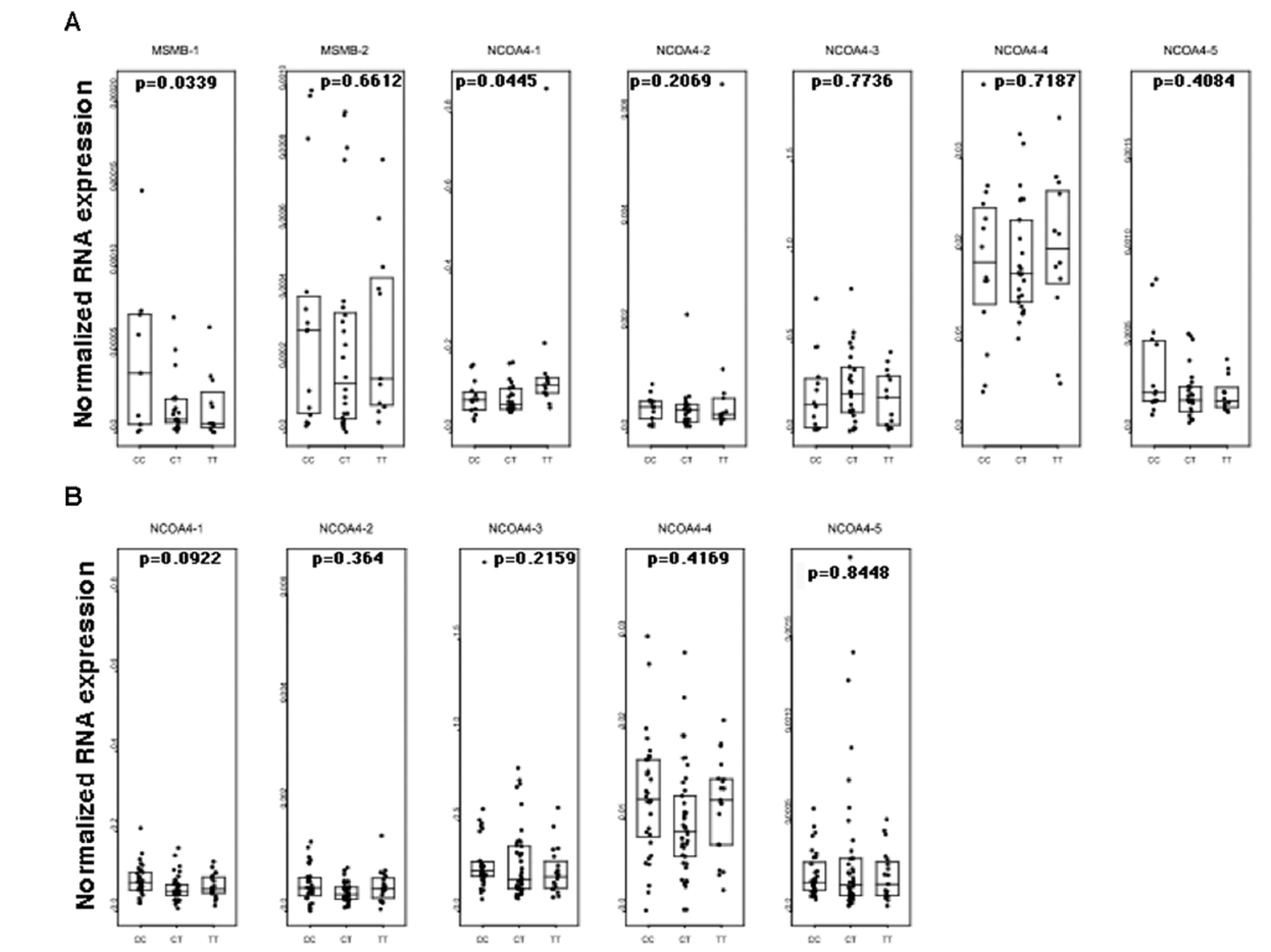

Supplement: Figure S3 — MSMB and NCOA4 expression in breast and colon epithelial tissue. Expression of MSMB and NCOA4 isoforms are not consistently associated with genotype at rs10993994 in breast or colon epithelial tissue. A. Expression in histologically normal breast tissue (n = 56). P-values for association between genotype and expression included within each graph. B. Expression in histologically normal colon tissue (n = 72). (9.47 MB TIF) [file pgen.1001204.s003.tif]

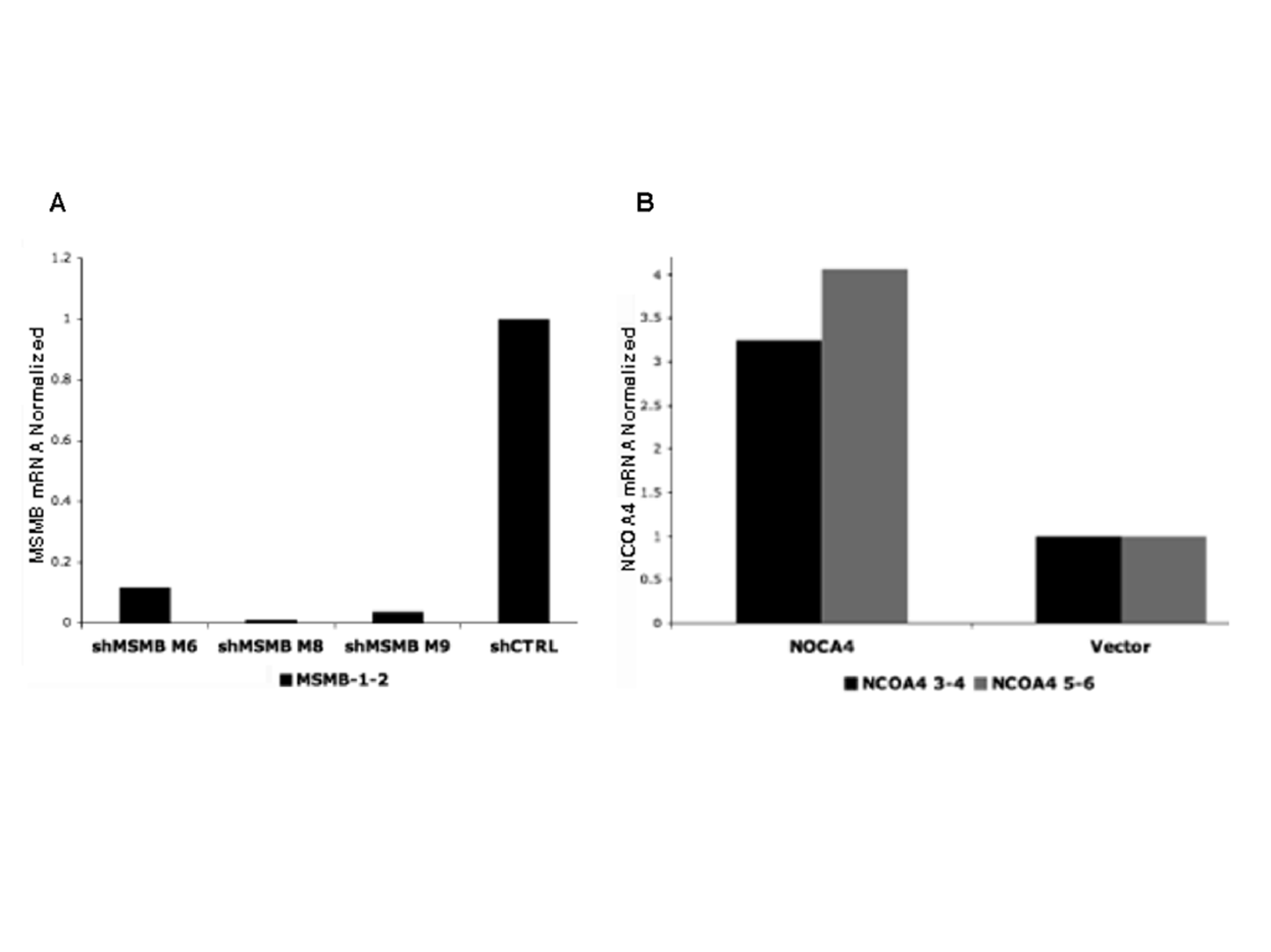

Supplement: Figure S4 — Quantitative RT-PCR for the expression of MSMB and NCOA4 in LHSAR cells. A. MSMB suppression with three individual shRNAs (shMSMB M6, M8 and M9). B. NCOA4 overexpression. (9.46 MB TIF) [file pgen.1001204.s004.tif]

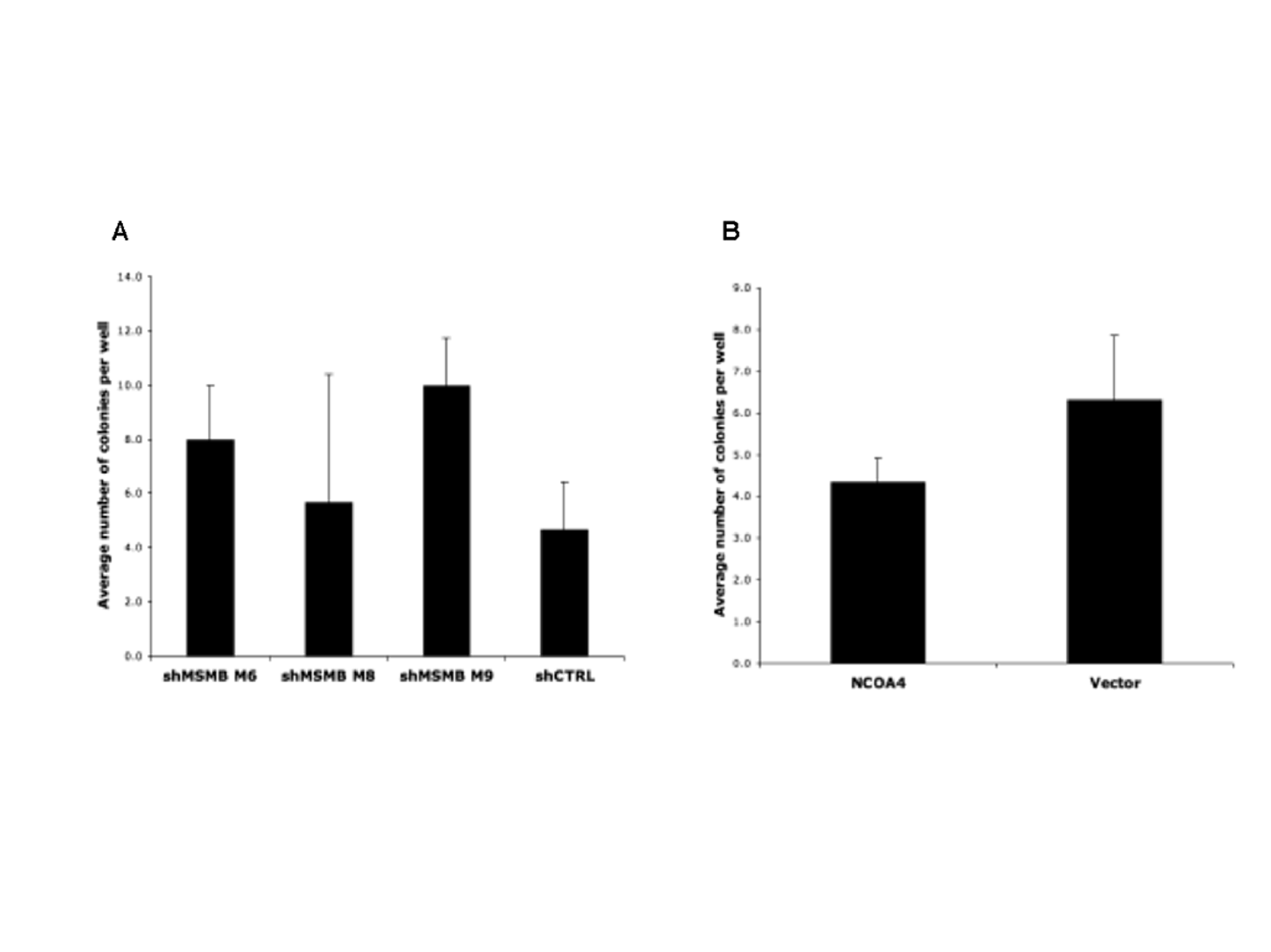

Supplement: Figure S5 — Anchorage-independent growth in human mammary epithelial cells. MSMB and NCOA4 expression levels do not consistently affect anchorage-independent growth of human mammary epithelial cells (HMLE). A. Suppression of MSMB by three independent shRNAs (p-values 0.0949, 0.7482, and 0.0197). B. NCOA4 overexpression (p = 0.1012). (9.46 MB TIF) [file pgen.1001204.s005.tif]
